# The Effect of SERM/CB_2_ Receptor Modulators on Repetitive Behaviours in Juvenile and Young Adult Mice May Have Implications for Tourette Syndrome Treatment

**DOI:** 10.3390/ijms27031181

**Published:** 2026-01-24

**Authors:** Victoria Gorberg, Peter McCaffery, Sharon Anavi-Goffer

**Affiliations:** Institute of Medical Sciences, University of Aberdeen, Aberdeen AB24 2ZD, UK; victoria.gorberg@abdn.ac.uk (V.G.);

**Keywords:** Tourette syndrome (TS), Tic disorder, CB_1_ receptor, CB_2_ receptor, endocannabinoid system, obsessive–compulsive disorder (OCD), selective oestrogen receptor modulator (SERM), clomiphene, Evista, raloxifene, bazedoxifene

## Abstract

Tourette syndrome (TS) is a neurodevelopmental disorder, with a male-to-female ratio of approximately 3:1, characterised by involuntary tics, frequently comorbid with conditions such as obsessive–compulsive disorder (OCD). Some patients exhibit limited responsiveness to standard medications, necessitating alternative therapeutic strategies. Clomiphene, a selective oestrogen receptor modulator (SERM), emerged as a potential candidate. However, raloxifene and bazedoxifene, which exhibit distinct chemical structures from clomiphene, present dual modulation not only as oestrogen receptor modulators but also as inverse agonists of the cannabinoid CB_2_ receptor. The present study compared the efficacy of clomiphene, raloxifene, and bazedoxifene in alleviating TS/OCD-like behaviours in mice. The findings revealed dose, sex, and age differences in the effects of raloxifene, and to a lesser extent of bazedoxifene, demonstrating potential therapeutic benefit for treating TS/OCD-like behaviours. The effects of raloxifene were compared in the presence of 2,5-dimethoxy-4-iodoamphetamine (DOI)-induced or SR141716A-induced motor-like tics, premonitory urges-induced, and OCD-like behaviours in mice. DOI-induced juvenile male and female mice responded to raloxifene, while only adolescent DOI-induced females responded to raloxifene. These results suggest that SERM drugs that are also CB_2_ receptor antagonists/inverse-agonists may be a new class of drugs to reduce motor tics and OCD symptoms in patients with TS/OCD.

## 1. Introduction

Tourette syndrome (TS) is a disorder of the nervous system that causes people (3:1 male/female) to have tics, which are involuntary twitches, movements, and sounds that cannot be controlled [1,2]. In 90% of the patients, TS co-exists with other conditions such as obsessive-compulsive behaviour (OCD) and hyperactivity [1,2]. Emerging evidence suggests that the endocannabinoid system (ECS) plays a role in the pathophysiology of obsessive–compulsive disorder (OCD). The ECS is expressed within cortico-striatal and cortico-limbic circuits and modulates synaptic activity in networks implicated in motor control, habit formation, and repetitive behaviours [3]. The ECS comprises two officially recognised receptors, the cannabinoid receptor type 1 (CB_1_) and cannabinoid receptor type 2 (CB_2_) (reviewed in [4]). Although CB_2_ receptors were initially thought to be absent from the central nervous system, functional expression has since been reported in several brain regions relevant to motor control, including the striatum, brainstem, cortex, and ventral tegmental area, where CB_2_-related signalling can influence dopaminergic and inhibitory neurotransmission [5,6]. Preclinical studies demonstrate that selective CB_2_ receptor ligands can modulate locomotor activity, highlighting a complex role for CB_2_ signalling in motor regulation [7,8,9,10,11]. In parallel, clinical studies indicate that cannabinoid-based treatments, including Δ^9^-tetrahydrocannabinol-derived medications, can ameliorate compulsive symptoms in subsets of patients with OCD [12,13,14,15]. Together, these findings suggest that ECS modulation, including CB_2_-related pathways, may represent a novel therapeutic target for Tourette syndrome and OCD.

In some patients, painful tics are unresponsive to the administration of the commonly prescribed medicines (e.g., haloperidol, clonidine), leading physicians to search for alternative therapeutics. One example is the use of clomiphene, a selective oestrogen-receptor modulator (SERM) prescribed for the treatment of female infertility. A clinical report described a reduction in both the severity and frequency of motor and phonic tics in an adult male patient with TS and OCD following one week of treatment with clomiphene (Clomid^®^; 25 mg twice a day) [16].

Since the introduction of clomiphene, a series of novel SERMs have been developed for the treatment of osteoporosis. [17]. The structure–activity relationship (SAR) of these compounds requires oestrogen-like efficacy in bone to reduce fracture incidence, while avoiding oestrogen-like stimulatory effects on uterine or mammary tissue (SAR is shown in Figure 3 of [17]). The first agent to fulfil this pharmacological profile was raloxifene, a benzothiophene derivative [17,18], followed by bazedoxifene, a third-generation SERM belonging to the indole family, in contrast to clomiphene, which belongs to the triphenylethylene family [17].

Interestingly, selected FDA/EMA-approved SERMs have been shown to inhibit the cannabinoid CB_2_ receptor signalling, thereby acting on both oestrogen receptor and CB_2_ pathways as dual modulators. [19,20,21]. Library screening of FDA-approved drugs identified raloxifene (Evista^®^) and bazedoxifene as novel CB_2_ receptor inverse agonists [20,21,22], whereas tamoxifen, a triphenylethylene SERM, was found to act as a CB_1_/CB_2_ receptor inverse agonist [19]. These findings suggest that the pharmacological profile of SERMs is more complex than previously understood.

Treatment with clomiphene in a male patient with TS/OCD was accompanied by alterations in plasma levels, which regulate the production of sex hormones (oestrogens, progestogens, androgens) [16]. This observation suggests a link between sex hormones, i.e., oestrogen, and the clinical expression of TS in males. Notably, the same patient who responded to clomiphene also exhibited a low plasma level of luteinising hormone (LH) [16]. Moreover, an interaction between cannabinoids and gonadotropins has been reported: endocannabinoids suppress the release of hormones of the hypothalamic–pituitary–gonadal axis, thereby reducing LH secretion [23]. In addition, CB_2_ receptors have been identified in hypothalamic gonadotropin-releasing hormone (GnRH)-releasing neurons [24].

Collectively, the evidence suggests that selected SERMs not only modulate oestrogen receptors but may also act on CB_2_ receptors, directly influencing motor control [10,25] and indirectly regulating the release of sex hormones via the hypothalamic-pituitary-gonadal axis [23,24], thereby supporting the hypothesis of hypothalamic involvement in TS [26].

Raloxifene has already been proposed for the treatment of central nervous system (CNS) disorders, including cognitive impairments (clinical trials), Parkinson’s disease, and multiple sclerosis (animal models) [27]. Therefore, characterising raloxifene and other selected FDA/EMA-approved SERMs in models of TS may facilitate their therapeutic repositioning and expand the spectrum of SERM/CB_2_ receptor inverse agonists as a potential new class of drugs for patients with TS. We have recently demonstrated a role for the CB_2_ receptor in TS, in which CB_2_ receptor stimulation enhances 2,5-dimethoxy-4-iodoamphetamine (DOI)-induced motor-like tics [25]. In this study, we compared the effects of raloxifene and bazedoxifene with those of clomiphene in the (DOI)-induced motor-like tic model. The results of this study suggest that novel SERMs with CB_2_ receptor antagonist/inverse-agonist properties may reduce motor tics and OCD symptoms in patients with TS/OCD.

## 2. Results

### 2.1. Effects of Clomiphene Citrate, Raloxifene, and Bazedoxifene on HTR

Clomiphene was clinically administered to treat one adult male patient with TS/OCD [16]. However, to the best of our knowledge, the efficacy of clomiphene, raloxifene, and bazedoxifene in animal models of TS has not been tested. Moreover, the onset of TS occurs in childhood; therefore, we evaluated the effect of clomiphene in 3-week-old mice. In addition, we evaluated the effects of each drug in the absence of a tic-like inducer. This may point to potential side effects in humans and may reflect on the therapeutic window of each drug, i.e., the absence or reduction in effects may predict a wider therapeutic window. Differences between doses may reflect potential therapeutic doses.

**Figure 1:**
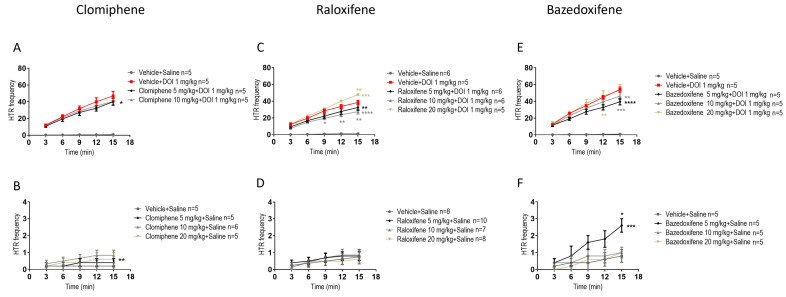
Effects of clomiphene citrate, raloxifene, and bazedoxifene on head twitch response (HTR) in juvenile male mice with or without DOI. Clomiphene citrate in the presence (**A**) and absence (**B**) of DOI; raloxifene in the presence (**C**) and absence (**D**) of DOI; and bazedoxifene in the presence (**E**) and absence (**F**) of DOI. Data are means ± SEM; n represents the number of 3-week-old C57BL/6J male mice per group. Experiments were independently repeated as required to achieve the lowest n. Statistical analysis: two-way ANOVA followed by Bonferroni’s post hoc test (* *p* = 0.05; ** *p* < 0.01; *** *p* < 0.001; **** *p* < 0.0001).

In juvenile male mice, compared to DOI, clomiphene at 5 mg/kg in the presence of DOI significantly decreased the DOI-induced HTR by 15% (Figure 1A; F (1, 40) = 4.595, *p* = 0.0382; two-way ANOVA), while clomiphene at 10 mg/kg had a similar effect, which did not reach a significant level. The effect of clomiphene, compared to its vehicle (control), in the absence of DOI, clomiphene at 10 mg/kg significantly increased HTR by 317% compared to vehicle (Figure 1B; F (1, 45) = 8.330, *p* = 0.0060; two-way ANOVA).

Recent studies have found that raloxifene and bazedoxifene, SERM drugs, are also CB_2_ receptor inverse agonists [20,22]. It was important to compare their effects with those of clomiphene. In juvenile male mice, compared to DOI, raloxifene at 5, 10 mg/kg in the presence of DOI significantly decreased the DOI-induced HTR by 16% and 29%, respectively (Figure 1C; F (1, 45) = 9.14, *p* = 0.0048, for 5 mg/kg; F (1, 45) = 33.96, *p* < 0.0001, for 10 mg/kg; two-way ANOVA). However, raloxifene at 20 mg/kg significantly increased DOI-induced HTR by 25% (Figure 1C; F (1, 40) = 15.35, *p* = 0.0003, for 20 mg/kg; two-way ANOVA). In contrast to clomiphene, in the absence of DOI, raloxifene at 5, 10, 20 mg/kg had no significant effect on HTR compared to its vehicle (control) (Figure 1D).

In juvenile male mice, compared to DOI, bazedoxifene at 5, 10 mg/kg in the presence of DOI significantly decreased the DOI-induced HTR by 28% and 15%, respectively (Figure 1E; F (1, 40) = 33.96, *p* < 0.0001, for 5 mg/kg; F (1, 40) = 8.930, *p* = 0.0048, for 10 mg/kg; two-way ANOVA). An important effect of bazedoxifene relative to its vehicle (control) was observed. Bazedoxifene at 5 mg/kg significantly increased HTR by 225% (Figure 1F; F (1, 40) = 14.22, *p* = 0.0005; two-way ANOVA).

### 2.2. Effects of Clomiphene Citrate, Raloxifene, and Bazedoxifene on ESR

**Figure 2:**
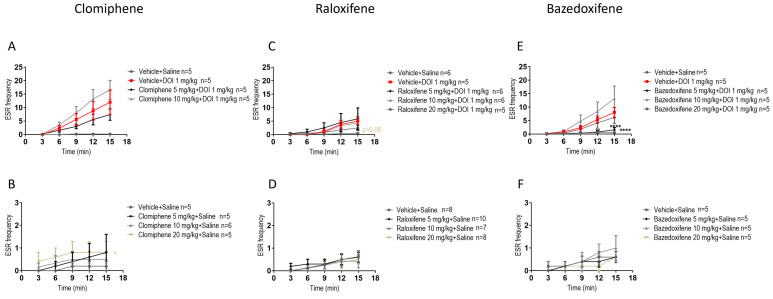
Effects of clomiphene citrate, raloxifene, and bazedoxifene on ear scratch response (ESR) in juvenile male mice with or without DOI. Clomiphene citrate in the presence (**A**) and absence (**B**) of DOI; raloxifene in the presence (**C**) and absence (**D**) of DOI; and bazedoxifene in the presence (**E**) and absence (**F**) of DOI. Data are means ± SEM; n represents the number of 3-week-old C57BL/6J male mice per group. Experiments were independently repeated as required to achieve the lowest n. Statistical analysis: two-way ANOVA followed by Bonferroni’s post hoc test (* *p* = 0.05; **** *p* < 0.0001).

In juvenile male mice, compared to DOI, clomiphene at 5, 10, 20 mg/kg in the presence of DOI had no significant effect on the DOI-induced ESR (Figure 2A). Compared to the vehicle (control), clomiphene citrate at 20 mg/kg significantly increased ESR by 300% (Figure 2B; F (1, 40) = 6.759, *p* = 0.0130; two-way ANOVA).

In juvenile male mice, compared to DOI, raloxifene at 5, 10, 20 mg/kg in the presence of DOI had no significant effect on the DOI-induced ESR (Figure 2C). Compared to the vehicle (control), raloxifene at 5, 10, and 20 mg/kg had no significant effect on ESR (Figure 2D).

An important effect on ESR was found with bazedoxifene. In juvenile male mice, compared to DOI, bazedoxifene at 5 mg/kg in the presence of DOI significantly decreased the DOI-induced ESR by 86% (Figure 2E; F (1, 40) = 25.39, *p* < 0.0001; two-way ANOVA). Compared to the vehicle (control), bazedoxifene at 5, 10, 20 mg/kg had no significant effect on ESR (Figure 2F).

### 2.3. Effects of Clomiphene Citrate, Raloxifene, and Bazedoxifene on Grooming Behaviour

**Figure 3:**
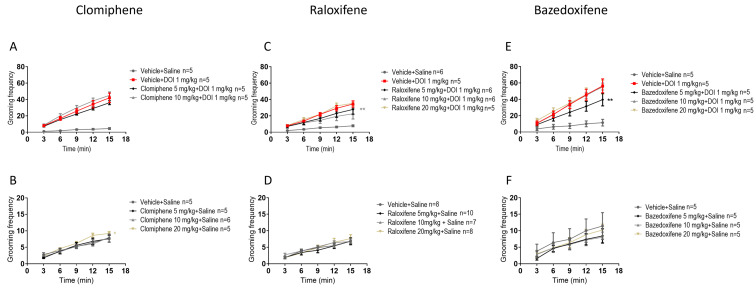
Effects of clomiphene citrate, raloxifene, and bazedoxifene on grooming behaviour in juvenile male mice with or without DOI. Clomiphene citrate in the presence (**A**) and absence (**B**) of DOI; raloxifene in the presence (**C**) and absence (**D**) of DOI; and bazedoxifene in the presence (**E**) and absence (**F**) of DOI. Data are means ± SEM; n represents the number of 3-week-old C57BL/6J male mice per group. Experiments were independently repeated as required to achieve the lowest n. Statistical analysis: two-way ANOVA followed by Bonferroni’s post hoc test (* *p* = 0.05; ** *p* < 0.01).

In juvenile male mice, compared with DOI, clomiphene at 5, 10, and 20 mg/kg in the presence of DOI had no significant effect on the DOI-induced grooming behaviour (Figure 3A). Compared to the vehicle (control), clomiphene at 20 mg/kg significantly increased grooming behaviour by 18% (Figure 3B; F (1, 40) = 4.571, *p* = 0.0387; two-way ANOVA).

In juvenile male mice, compared to DOI, raloxifene at 10 mg/kg in the presence of DOI significantly decreased the DOI-induced grooming behaviour by 45% (Figure 3C; F (1, 45) = 7.345, *p* = 0.0095; two-way ANOVA). Importantly, compared to the vehicle (control), raloxifene at 5, 10, and 20 mg/kg had no significant effect on grooming behaviour (Figure 3D).

In juvenile male mice, compared to DOI, bazedoxifene at 5 mg/kg in the presence of DOI significantly decreased DOI-induced grooming behaviour by 37% (Figure 3E; F (1, 40) = 7.494, *p* = 0.0092; two-way ANOVA). Compared to the vehicle (control), bazedoxifene at 5, 10, 20 mg/kg had no significant effect on grooming behaviour (Figure 3F).

Across all experiments, there was no significant difference in body weight between the groups in the presence (Supplementary Figure S1A,C,E) or absence (Supplementary Figure S1B,D,F) of DOI.

### 2.4. Effects of Raloxifene on DOI-Induced Locomotor Activity in Juvenile Male Mice

Compared with clomiphene and bazedoxifene, raloxifene appeared to have a greater inhibitory effect on DOI-induced repetitive behaviours in juvenile mice. In addition, although bazedoxifene had similar inhibitory effects to those of raloxifene, bazedoxifene at 5 mg/kg significantly increased HTR in untreated mice (i.e., in the absence of DOI), suggesting that it could produce side effects in patients with central tics. Therefore, we have focused our investigation on the effects of raloxifene.

We have previously shown that in healthy juvenile male mice, HU-308, a CB_2_ receptor selective agonist, increases motor-like tics while reducing locomotor activity in the open field test [10]. Compared with HU-308, in healthy juvenile male mice, raloxifene at 10 mg/kg had no effect on ambulation, rearing, jumping, and grooming behaviours (Supplementary Figure S2A–D, respectively) nor on faeces number (Supplementary Figure S2E).

In contrast, DOI significantly increased ambulation, rearing, jumping, and grooming behaviours (Supplementary Figure S2A–D), but did not affect faeces number (Supplementary Figure S2E). Compared with the inhibitory effects of raloxifene on repetitive behaviours, raloxifene at 10 mg/kg did not affect DOI-induced ambulation, rearing, jumping, and grooming behaviours or faeces number in juvenile male mice (Supplementary Figure S2A–E, respectively). Body weight between groups was similar (Supplementary Figure S2F).

We have previously shown the effect of ∆^9^-THC (1 mg/kg) on HTR, ESR, and grooming behaviours in juvenile mice [11], but not in the open field test. Similarly to raloxifene, in juvenile males, ∆^9^-THC at 1 mg/kg had no effect on ambulation, rearing, or grooming behaviours, nor on DOI-induced ambulation, rearing, or grooming behaviours (Supplementary Figure S3A,B,D). However, ∆^9^-THC (1 mg/kg) reversed the DOI-induced jumping behaviour (Supplementary Figure S3C) while raloxifene did not (Supplementary Figure S2C). Further neurological studies may provide important information about the apparent distinct origins of neuronal populations that lead to grooming vs. jumping peripheral motor-like tics.

### 2.5. Effects of Raloxifene on DOI-Induced Repetitive Behaviours in Juveniles: Sex Differences

**Figure 4:**
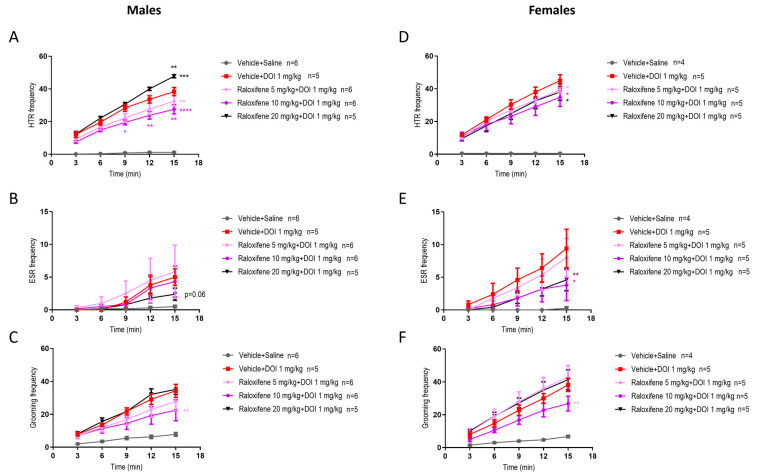
Effects of raloxifene on DOI-induced HTR, ESR, and grooming behaviour in juvenile male and female mice. The effects of raloxifene on HTR in juvenile male (**A**) and female (**D**) mice; ESR in male (**B**) and female (**E**) mice; grooming in male (**C**) and female (**F**) mice. Data are means ± SEM; n = C57BL/6J mice per group. Experiments were independently repeated to the lowest n. Two-way ANOVA with Bonferroni’s post hoc test. (* *p* = 0.05; ** *p* < 0.01; *** *p* < 0.001; **** *p* < 0.0001).

As raloxifene was designed to treat adult females, it was important to investigate if there were sex differences. In juvenile male mice, raloxifene at 5 and 10 mg/kg in the presence of DOI reduced the DOI-induced HTR by 16% and 29%, respectively (Figure 1C and Figure 4A), whereas in juvenile females, the reductions were 12% and 23%, respectively (Figure 4D; F (1, 40) = 6.140, *p* = 0.0175 for 5 mg/kg; F (1, 40) = 6.758, *p* = 0.0130 for 10 mg/kg; two-way ANOVA). However, in juvenile male mice, raloxifene at 20 mg/kg significantly increased the DOI-induced HTR by 25% (Figure 1C and Figure 4A). In contrast, in juvenile females, raloxifene at 20 mg/kg significantly decreased the DOI-induced HTR by 16% (Figure 4D; F (1, 40) = 5.243, *p* = 0.0274; two-way ANOVA).

In juvenile male mice, raloxifene at 5, 10, and 20 mg/kg in the presence of DOI had no significant effect on the DOI-induced ESR (Figure 2C and Figure 4B). In contrast, in juvenile female mice, raloxifene at 10 and 20 mg/kg in the presence of DOI significantly decreased the DOI-induced ESR by 61% and 52%, respectively (Figure 4E; F (1, 40) = 6.088, *p* = 0.0180, for 10 mg/kg; F (1, 40) = 7.556, *p* = 0.0089, for 20 mg/kg; two-way ANOVA).

In juvenile male mice, raloxifene at 10 mg/kg in the presence of DOI significantly decreased the DOI-induced grooming behaviour by 45% (Figure 3C and Figure 4C), whereas in juvenile female mice raloxifene at 10 mg/kg in the presence of DOI significantly decreased the DOI-induced grooming behaviour by 36% (Figure 4F; F (1, 40) = 11.03, *p* = 0.0019; two-way ANOVA).

**Figure 5:**
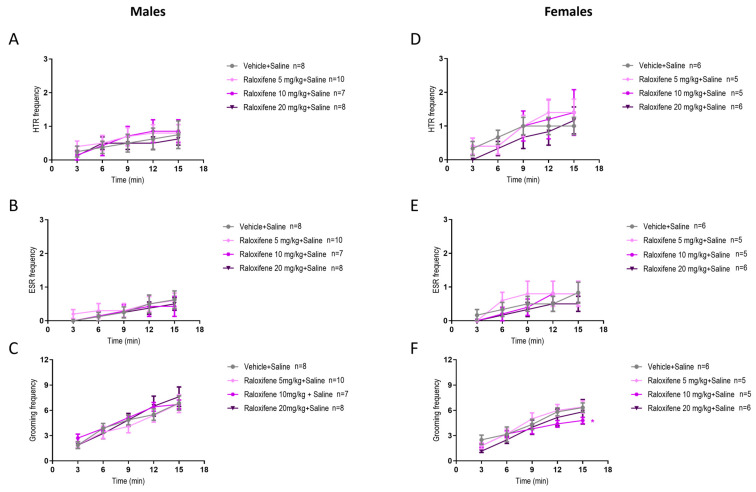
Effects of raloxifene on HTR, ESR, and grooming behaviour in juvenile male and female mice in the absence of DOI. The effects of raloxifene on HTR in juvenile male (**A**) and female (**D**) mice; ESR in male (**B**) and female (**E**) mice; grooming in male (**C**) and female (**F**) mice. Data are means ± SEM; n = C57BL/6J mice per group. Experiments were independently repeated to the lowest n. Two-way ANOVA with Bonferroni’s post hoc test. (* *p* = 0.05).

In healthy juvenile male and females mice (i.e., in the absence of DOI), compared to the vehicle (control), raloxifene at 5, 10, 20 mg/kg did not affect HTR (Figure 5A and Figure 5D, respectively), ESR (Figure 5B and Figure 5D, respectively) or grooming behaviour (Figure 5C and Figure 5F, respectively), apart from a significant reduction at 10 mg/kg of grooming behaviour in juvenile female mice by 24% (Figure 5F; F (1, 45) = 5.590, *p* = 0.0224; two-way ANOVA).

There was no significant difference in the body weight of juvenile mice between the groups in the presence (Supplementary Figure S4A,C) or absence (Supplementary Figure S4B,D) of DOI.

### 2.6. Effects of Raloxifene on SR141716A-Induced Repetitive Behaviours in Juveniles: Sex Differences

Similarly to DOI, in juvenile male mice, SR141716A (10 mg/kg) induces motor-like tics and premonitory urge-like behaviour in juveniles [10,28,29]. We have recently shown that these effects are inhibited by HU-308, a highly selective CB_2_ receptor agonist [10].

**Figure 6:**
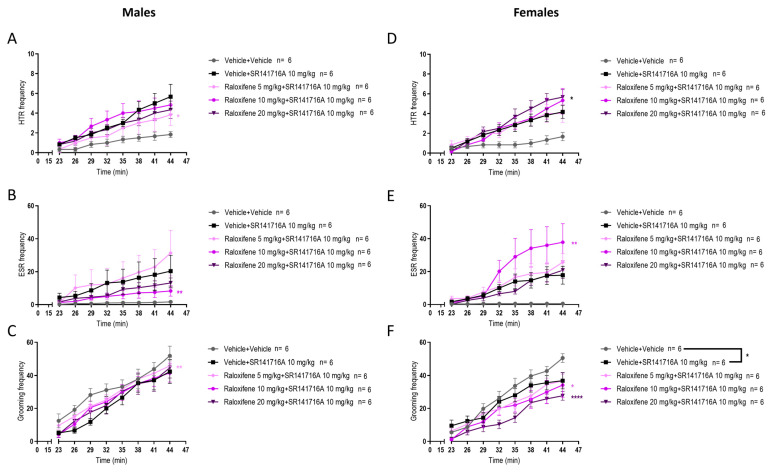
Effects of raloxifene on SR141716A-induced HTR, ESR, and grooming behaviour in juvenile male and female mice. The effects of raloxifene on HTR in juvenile male (**A**) and female (**D**) mice; ESR in male (**B**) and female (**E**) mice; grooming in male (**C**) and female (**F**) mice. Data are means ± SEM; n = C57BL/6J mice per group. Experiments were independently repeated to the lowest n. Two-way ANOVA with Bonferroni’s post hoc test. (* *p* = 0.05; ** *p* < 0.01; **** *p* < 0.0001).

In juvenile male mice, compared to SR141716A, raloxifene at 5 mg/kg in the presence of SR141716A significantly decreased the SR141716A-induced HTR by 48% (Figure 6A; F (1, 80) = 5.458, *p* = 0.0220; two-way ANOVA). Compared to SR141716A, raloxifene at 10 mg/kg in the presence of SR141716A significantly decreased the SR141716A-induced ESR by 64% (Figure 6B; F (1, 80) = 7.281, *p* = 0.0085; two-way ANOVA). Compared to the vehicle, SR141716A significantly reduced grooming behaviour (Figure 6C; F (1, 80) = 16.98, *p* < 0.000; two-way ANOVA). Compared to SR141716A, raloxifene at 5 mg/kg in the presence of SR141716A significantly increased the SR141716A-induced grooming behaviour by 37% (Figure 6C; F (1, 80) = 8.358, *p* = 0.0049; two-way ANOVA). However, this increase did not exceed the basal level of the vehicle group (Figure 6C).

In juvenile females, compared to SR141716A, raloxifene at 20 mg/kg significantly increased the SR141716A-induced HTR by 60% (Figure 6D; F (1, 80) = 5.074, *p* = 0.027; two-way ANOVA). Compared to SR141716A, raloxifene at 10 mg/kg in the presence of SR141716A significantly increased the SR141716A-induced ESR by 115% (Figure 6E; F (1, 80) = 9.981, *p* = 0.0022; two-way ANOVA). Compared to the vehicle, SR141716A significantly reduced grooming behaviour in juvenile females (Figure 6F; F (1, 80) = 4.737, *p* = 0.0325; two-way ANOVA). Compared to SR141716A, raloxifene at 10 and 20 mg/kg in the presence of SR141716A significantly reduced the SR141716A-induced grooming behaviour by 20% and 67%, respectively (Figure 6F; F (1, 80) = 5.369, *p* = 0.0231, for 10 mg/kg; F (1, 80) = 35.13, *p* < 0.0001, for 20 mg/kg; two-way ANOVA). These results indicate that raloxifene has sex-dependent effects in juvenile mice and that raloxifene at 20 mg/kg may be efficacious in reducing ‘peripheral’ motor tics in girls.

**Figure 7:**
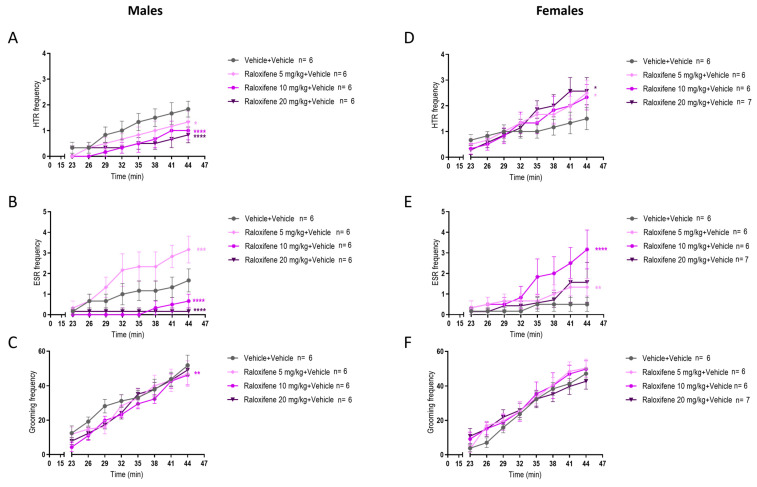
Effects of raloxifene on HTR, ESR, and grooming behaviour in juvenile male and female mice in the absence of SR141716A. The effects of raloxifene on HTR in juvenile male (**A**) and female (**D**) mice; ESR in male (**B**) and female (**E**) mice; grooming in male (**C**) and female (**F**) mice. Data are means ± SEM; n = C57BL/6J mice per group. Experiments were independently repeated to the lowest n. Two-way ANOVA with Bonferroni’s post hoc test. (* *p* = 0.05; ** *p* < 0.01; *** *p* < 0.001; **** *p* < 0.0001).

In healthy juvenile male mice (i.e., in the absence of tic-like inducer), compared to the vehicle (control), raloxifene at 5, 10, 20 mg/kg significantly decreased the HTR by 27%, 45% and 54%, respectively (Figure 7A; F (1, 80) = 5.664, *p* = 0.0197 for 5 mg/kg; F (1, 80) = 19.30, *p* < 0.0001 for 10 mg/kg; F (1, 80) = 19.23, *p* < 0.0001 for 20 mg/kg; two-way ANOVA). Compared to the vehicle (control), raloxifene at 5 mg/kg significantly increased the ESR by 90% (Figure 7B; F (1, 80) = 12.32, *p* = 0.0007; two-way ANOVA), but raloxifene at 10, 20 mg/kg significantly decreased the ESR by 60% and 90%, respectively (Figure 7B; F (1, 80) = 21.49, *p* < 0.0001 for 10 mg/kg; F (1, 80) = 24.14, *p* < 0.0001 for 20 mg/kg; two-way ANOVA). Compared to the vehicle (control), raloxifene at 10 mg/kg significantly decreased the grooming behaviour by 11% (Figure 7C; F (1, 80) = 9.585, *p* = 0.0027; two-way ANOVA).

In healthy juvenile female mice (i.e., in the absence of tic-like inducer), compared to the vehicle (control), raloxifene at 5, 20 mg/kg significantly increased the HTR by 67% and 71%, respectively (Figure 7D; F (1, 80) = 5.000, *p* = 0.0281, for 5 mg/kg; F (1, 88) = 5.807, *p* = 0.018 for 20 mg/kg; two-way ANOVA). Compared to the vehicle (control), raloxifene at 5 and 10 mg/kg significantly increased the ESR by 167% and 533% (Figure 7E; F (1, 80) = 8.672, *p* = 0.0042 for 5 mg/kg; F (1, 80) = 19.65, *p* < 0.0001 for 10 mg/kg; two-way ANOVA), but raloxifene at 20 mg/kg had no significant effect on ESR (Figure 7E). Compared to the vehicle (control), raloxifene at 5, 10, and 20 mg/kg did not affect grooming behaviour in juvenile females (Figure 7F).

### 2.7. Effects of Raloxifene on Repetitive Behaviours in Adolescent Mice: Sex Differences

As raloxifene appeared to inhibit DOI-induced repetitive behaviours in juveniles (Figure 4), it was important to compare its effects with those in adolescent (6-week-old) mice (Supplementary Figure S5).

In adolescent male mice, compared to DOI, raloxifene at 5, 20 mg/kg in the presence of DOI significantly decreased the DOI-induced HTR by 10% and 13%, respectively (Supplementary Figure S5A, F (1, 30) = 4.691, *p* = 0.0384, for 5 mg/kg; F (1, 30) = 5.058, *p* = 0.0320, for 20 mg/kg; two-way ANOVA) and raloxifene 5, 10, 20 mg/kg had no effect on DOI-induced ESR or on DOI-induced grooming behaviour (Supplementary Figure S5B,C). Moreover, in healthy adolescent male mice, compared to the vehicle (control), raloxifene at 5, 10 mg/kg significantly increased HTR by 500% and 599%, respectively (Supplementary Figure S6A; F (1, 20) = 16.33, *p* = 0.0006, for 5 mg/kg; F (1, 20) = 60.50, *p* < 0.0001, for 10 mg/kg; two-way ANOVA). These results suggest that in adolescent boys, raloxifene may have a negligible clinical significance effect on ‘central’ motor tics and may not have any effect on ‘peripheral’ motor tics.

In adolescent female mice, compared to DOI, raloxifene at 5 mg/kg in the presence of DOI significantly decreased the DOI-induced HTR by 21% (Supplementary Figure S5D; F (1, 30) = 6.301, *p* = 0.0177; two-way ANOVA). Compared to DOI, raloxifene at 10 mg/kg in the presence of DOI significantly increased the DOI-induced ESR by 66% (Supplementary Figure S5E; F (1, 30) = 6.570, *p* = 0.0156; two-way ANOVA). In adolescent female mice, compared to DOI, raloxifene at 5 mg/kg in the presence of DOI significantly decreased the DOI-induced grooming behaviour by 37% (Supplementary Figure S5F; F (1, 30)= 14.99, *p* = 0.0005; two-way ANOVA). These findings highlight the importance of further investigation into the potential clinical effects of raloxifene in adolescent girls.

In adolescent female mice, in the absence of DOI, compared to the vehicle (control), raloxifene at 5, 10, 20 mg/kg had no significant effect on HTR nor on ESR (Supplementary Figure S6D,E) and raloxifene at 5, 20 mg/kg significantly decreased grooming behaviour by 33% and 29% (Supplementary Figure S6F; F (1, 30) = 5.944, *p* = 0.0209, for 5 mg/kg; F (1, 30) = 5.915, *p* = 0.0212, for 20 mg/kg; two-way ANOVA). However, raloxifene at 10 mg/kg significantly increased grooming behaviour by 24% (Supplementary Figure S6F; F (1, 30) = 5.435, *p* = 0.0266, for 10 mg/kg; two-way ANOVA).

There was no significant difference in the body weight of adolescent male (Supplementary Figure S7A,B) or female (Supplementary Figure S7C,D) mice between the groups, regardless of the presence (Supplementary Figure S7A,C) or absence of DOI (Supplementary Figure S7B,D).

## 3. Discussion

### 3.1. Clomiphene Citrate, Raloxifene or Bazedoxifene for Repetitive Behaviour?

Following reports of the SERM clomiphene showing effects in patients with TS and the discovery that several SERMs act as novel CB_2_ receptor inverse agonists [20,22], we examined clomiphene, raloxifene, and bazedoxifene in TS models, focusing on motor-like tics in juvenile males. At 5 mg/kg, clomiphene moderately attenuated DOI-induced ‘central’ motor-like tics and showed no benefit for urge-like behaviour or peripheral motor-like tics. In healthy mice, clomiphene by itself induced spontaneous central and ‘peripheral’ motor-like tics and urges at the higher doses tested (10, 20 pmg/kg), highlighting dose-dependent effects. When considering human equivalent dose scaling, this finding raises the possibility that higher doses increase the risk of tic exacerbation in boys. This may explain why the use of clomiphene (Clomid^®^; 25 mg twice a day; t_1/2_ of 5–6 days) has not become an established therapy for TS motor tics [16].

In contrast, raloxifene produced a stronger inhibitory effect on DOI-induced repetitive behaviours in juvenile males, with significant reductions observed at 5 and 10 mg/kg. At 20 mg/kg, however, raloxifene increased repetitive behaviours, indicating a loss of therapeutic efficacy at higher doses. Notably, no significant effects were observed in the untreated mice. These findings suggest that raloxifene, at doses equivalent to 0.4–1.6 mg/kg HED, emerged as a promising lead compound for further preclinical and clinical evaluation in treating motor tics in boys with TS.

Bazedoxifene showed a more complex profile. At 5 mg/kg, it significantly reduced both ‘central’ and ‘peripheral’ DOI-induced motor-like tics and urge-like behaviours; in healthy mice, it evoked spontaneous central tics; and at 10 mg/kg, it exacerbated urges. These results suggest that bazedoxifene may not be suitable for tic suppression in boys and could even worsen central tics. However, when premonitory urges are predominant, a low HED (0.4 mg/kg) might reduce their frequency.

Notably, raloxifene and bazedoxifene, later-generation SERMs, significantly reduced DOI-induced grooming behaviour, representing ‘peripheral’ motor tics, by 37–45% without affecting grooming behaviour in untreated mice. This selective effect demonstrates superior efficacy to clomiphene in suppressing ‘peripheral’ tics and highlights their potential as more effective therapeutic candidates.

### 3.2. Age and Sex Differences

Age- and sex-dependent effects were also apparent. In young adult males, raloxifene at 5 mg/kg induced spontaneous ‘central’ motor-like tics and urges, whereas in young adult females, it reduced both ‘central’ and ‘peripheral’ DOI-induced tics without increasing them in untreated mice (i.e., healthy mice). These results suggest that raloxifene (0.4 mg/kg HED) may represent a potential treatment candidate for further study in young adult women with TS, but could exacerbate symptoms in young adult men. Similarly, at 10 mg/kg, raloxifene reduced motor-like tics in juvenile males but not urges, while in juvenile females it reduced both motor-like tics and urges and, importantly, suppressed spontaneous peripheral tics.

## 4. Materials and Methods

### 4.1. Animals

The experimental procedures described below were approved by the Institutional Animal Use and Care Committee of Ariel University and the University of Aberdeen in accordance with the UK Home Office, EU directive 63/2010E, and the Animal (Scientific Procedures) Act 1986.

The effects of selected SERMs were screened in C57BL/6J mice, the same strain employed in our previous studies investigating the actions of Δ^9^-THC and CBD [11] and HU-308, a highly selective CB_2_ receptor agonist [25]. For experiments conducted in the presence or absence of DOI, male and female C57BL/6J (OlaHsd sub-strain) mice were obtained from Envigo, Ness Ziona, Israel. For experiments conducted in the presence or absence of SR141716A, C57BL/6J mice were obtained from the animal facility at the University of Aberdeen, UK. For each purchased group of animals, one additional animal was included at the time of purchase to ensure continuity of the study in the event of unforeseen issues affecting litter viability. In cases where all animals remained healthy and suitable for experimentation, the additional animal was randomly assigned to an experimental group, which occasionally resulted in unequal group sizes. Animals were housed in a 12:12 h light–dark cycle at 24 °C, with ad libitum access to food and water. Experiments were performed in 3-week-old unweaned juvenile males and females, as well as in 6-week-old pubertal young adult males and females. For each experimental set, mice were group-housed 4–8 animals per cage and allowed to habituate to the housing environment for a minimum of one week prior to experimental procedures. All mice were included in the experiments unless atypical development or health-related concerns were observed, such as eye abnormalities or abnormalities affecting the tail or feet.

### 4.2. Drugs

(R)(−)-DOI hydrochloride (CAS 82864-02-6), dimethyl sulfoxide (DMSO), and Kolliphor^®^ EL were obtained from Merck KGaA (Israel). DOI (1 mg/kg) was dissolved in sterile 0.9% NaCl saline solution. Raloxifene hydrochloride (CAS 82640-04-8), bazedoxifene acetate (CAS PZ0018), and clomiphene citrate (CAS 50-41-9) were obtained from Merck (USA). Raloxifene, bazedoxifene, and clomiphene (5, 10, or 20 mg/kg) were dissolved in a vehicle solution consisting of DMSO, Cremophor^®^ EL, and sterile saline in a ratio of 0.6:1:18.4, respectively. SR141716A was synthesised by Dr Iain R. Greig, University of Aberdeen, UK (according to US Patent 5462960) [25]. SR141716A (10 mg/kg) was dissolved in the same vehicle solution as above.

All drugs were freshly prepared, aliquoted, and stored at −20 °C for up to 3 months, with each aliquot discarded after a single use. Control animals received the appropriate vehicle without the active compound. Compounds or vehicles were administered intraperitoneally (i.p.) at an injection volume of 10 μL/g body weight.

### 4.3. Measurement of Head Twitch Response (HTR), Ear Scratch Response (ESR), and Grooming Behaviour

**Figure 8:**
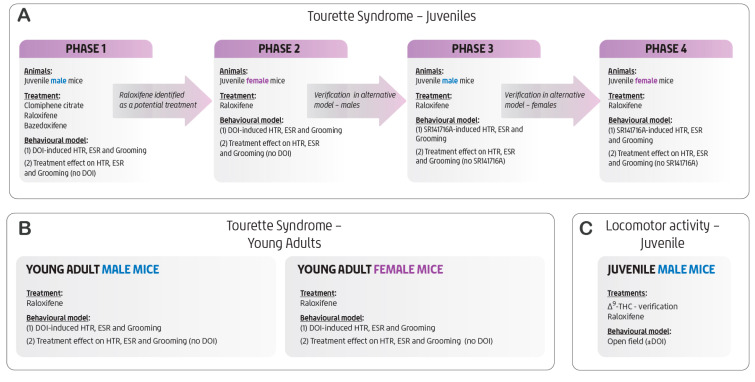
Schematic outline of the study phases. Behavioural models used to induce Tourette syndrome–like behaviours in juvenile (**A**) and young adult (**B**) mice. Additional data showing the effects of raloxifene and Δ9-THC, with and without DOI, on locomotor activity (**C**).

Details of the DOI model system, including experimental procedures, model limitations, and the approaches to blinding and randomisation, have been reported in our earlier work [11,25]. The present study focused on the acute effects of SERMs rather than on longer-term exposure or potential hormonally mediated changes. The study was conducted in phases, as outlined in Figure 8. Briefly, groups of 4–8 mice were housed per cage and habituated to the experimental environment for 60 min in their home cage prior to testing. For each model system, doses were randomised within each experimental set, and mice were tested in random order. Each experimental set, comprising appropriate controls and varying concentrations of a given SERM with or without DOI or SR141716A (as specified in the figure legends), was conducted within a single day. The experimenter was semi-blinded to the treatment groups. Each mouse received a randomised intraperitoneal (i.p.) injection of either the test compound or its vehicle. The first injection was followed 60 min later with a second injection (i.p.) of DOI (1 mg/kg) or saline, as its vehicle, for the DOI-induced model system; or of SR141716A (10 mg/kg) or its vehicle (DMSO, Cremophor^®^ EL, sterile saline as documented above) for the SR141716A-induced model system. Each mouse was tail-marked for identification and immediately placed in the centre of a clear glass cage (30 × 40 × 30 cm). Behavioural scoring began five minutes later for the DOI-induced model system and 20 min later for the SR141716A-induced model system. The number of head-twitch responses (HTRs), ear-scratch responses (ESRs), and grooming episodes was recorded over a 15-min period for the DOI-induced model system and 24 min for the SR141716A-induced model system in three-minute intervals for each mouse. HTR was counted every time the mouse had a head twitch. ESR was counted each time the mouse scratched itself with its hind limbs. Self-grooming was counted each time the mouse groomed any body part with its forelimbs or hind limbs, or licked and cleaned the tail or nails. To avoid overcounting, a new ESR or grooming action was only added to the total if the mouse had moved all four paws since the preceding action [10,11].

### 4.4. Statistical Analysis

All data were expressed as a mean ± SEM. *p <* 0.05 was considered statistically significant. Data were analysed with GraphPad Prism version 9 (GraphPad, San Diego, CA, USA). Line curves of HTR, ESR, grooming, ambulation, and rearing behaviours were analysed by two-way analyses of variance (ANOVA) followed by the Bonferroni *post hoc* test. *post hoc* tests were performed only when the F ratio was significant, as indicated below (*p <* 0.05). The frequency percentages of HTR, ESR, and grooming behaviour were calculated as previously described [11].

## 5. Conclusions

Taken together, novel FDA/EMA-approved SERM drugs that are also CB_2_ receptor antagonists/inverse-agonists may be a new class of drugs capable of reducing central and peripheral motor tics in patients with TS/OCD. These results highlight the therapeutic potential of SERMs, particularly raloxifene, as a possible first-in-class CB_2_ targeting agent for TS/OCD. Raloxifene may provide therapeutic benefit for boys and girls with motor tics, with additional efficacy against urges in girls. The age- and sex-specific responses observed underline the importance of tailored approaches in developing such therapies for the treatment of motor tics. Further research will be needed to continue characterising the effects of these compounds and to assess their potential for future therapeutic strategies for Tourette syndrome.

## Data Availability

The data that support the findings of this study are available from the corresponding author upon reasonable request. Some data may not be made available because of privacy or ethical restrictions.

## Supplementary Materials

The following supporting information can be downloaded at: https://www.mdpi.com/article/10.3390/ijms27031181/s1.

## Abbreviations

The following abbreviations are used in this manuscript:

TS	Tourette syndrome
OCD	Obsessive–compulsive disorder
ESR	Ear scratch response
HTR	Head twitch response
∆^9^-THC	∆^9^-tetrahydrocannabinol
DOI	2,5-dimethoxy-4-iodoamphetamine
CB_1_	Cannabinoid type 1
CB_2_	Cannabinoid type 2
FDA	Food and Drug Administration
EMA	European Medicines Agency
DMSO	Dimethyl sulfoxide
