# Supplementary material for: The Effect of SERM/CB2 Receptor Modulators on Repetitive Behaviours in Juvenile and Young Adult Mice May Have Implications for Tourette Syndrome Treatment
Source: Int J Mol Sci. 2026 Jan 24;27(3):1181. doi: 10.3390/ijms27031181 (PMC12898493; doi:10.3390/ijms27031181)
Supplement: Supplementary file 1 [file ijms-27-01181-s001.zip › ijms-4044625-supplementary.pdf]

International Journal of *Molecular Sciences*

MDPI

Supplementary Material

Supplementary Material and Methods

Drugs

Δ^9-THC (98%) was kindly provided by Prof. Mechoulam (The Hebrew University, Israel). Δ^9-THC (5 mg/kg) was dissolved in a vehicle made of 0.6:1:1.84 DMSO/Kolliphor® EL/saline.

Open Field Test

The test was performed similarly to the methods previously described (Hanus et al., 1999) and is detailed in the 'Supplementary Information' of our previous study (Gorberg et al., 2022). Briefly, mice were habituated in their home cage for 60 min, then received an injection of a SERM or vehicle and were returned to the cage. After 60 min, each mouse received a second injection with DOI or Saline and was immediately placed in the centre of a clear glass experimental cage (30 × 30 × 30 cm) marked into a 4 × 4 grid. Ambulation, rearing, and grooming behaviours were manually scored. Ambulation was defined as crossing a grid line with all four paws; rearing as standing on the hindlimbs with forepaws extended (on the wall or in the air, excluding grooming); and grooming as forepaw grooming of the face, body, tail, or nails. Grooming was recorded continuously for 8 min, whereas ambulation and rearing were scored at 2-min intervals.

Statistical Analysis

All data were expressed as a mean ± SEM. $p < 0.05$ was considered statistically significant. Data were analysed with GraphPad Prism version 9 (GraphPad, San Diego, CA, USA). Line curves of HTR, ESR, grooming, ambulation, and rearing behaviours were analysed by two-way analyses of variance. ANOVA was followed by the Bonferroni *post hoc* test. *Post hoc* tests were performed only when the F ratio was significant ($p < 0.05$), as indicated below. The frequency percentages of HTR, ESR, and grooming behaviour were calculated as previously described (Gorberg et al., 2021).

Supplementary Figures

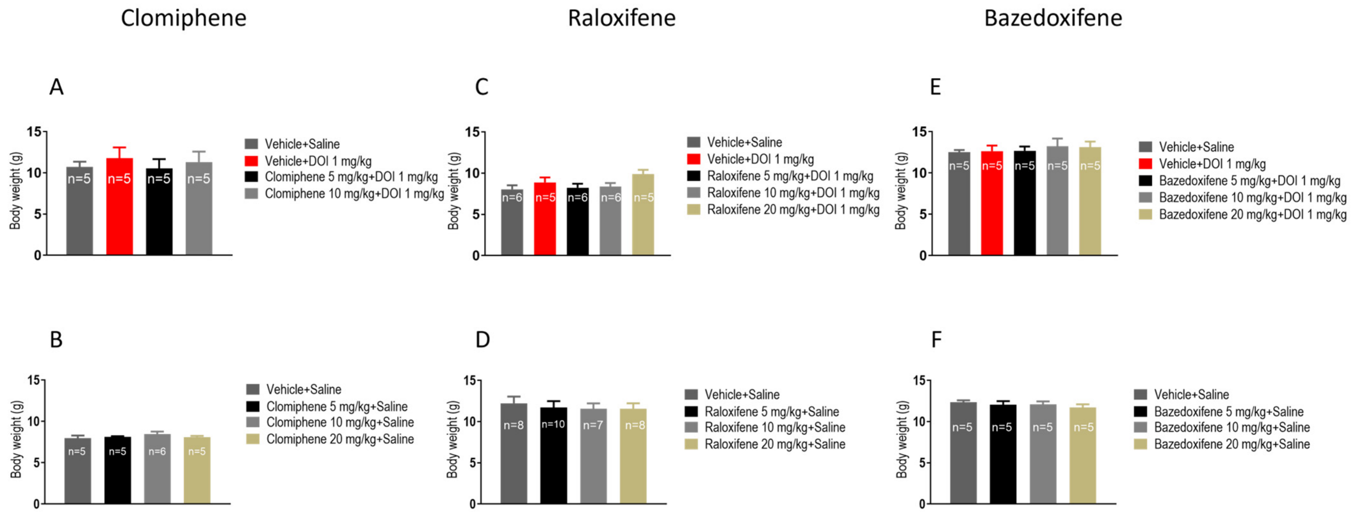

Figure S1. Effect of clomiphene citrate, raloxifene, and bazedoxifene on body weight in juvenile mice in the presence and absence of DOI. The effects of clomiphene citrate in the presence (**A**) and absence (**B**) of DOI. The effects of raloxifene in the presence (**C**) and absence (**D**) of DOI. The effects of bazedoxifene in the presence (**E**) and absence (**F**) of DOI. Data shown are means ± SEM; n represents the number of 3-week-old C57BL/6J male mice in each group. The experiment was independently repeated a number of times according to the lowest n. Two-way ANOVA analysis of variance followed by Bonferroni's test for multiple comparisons.

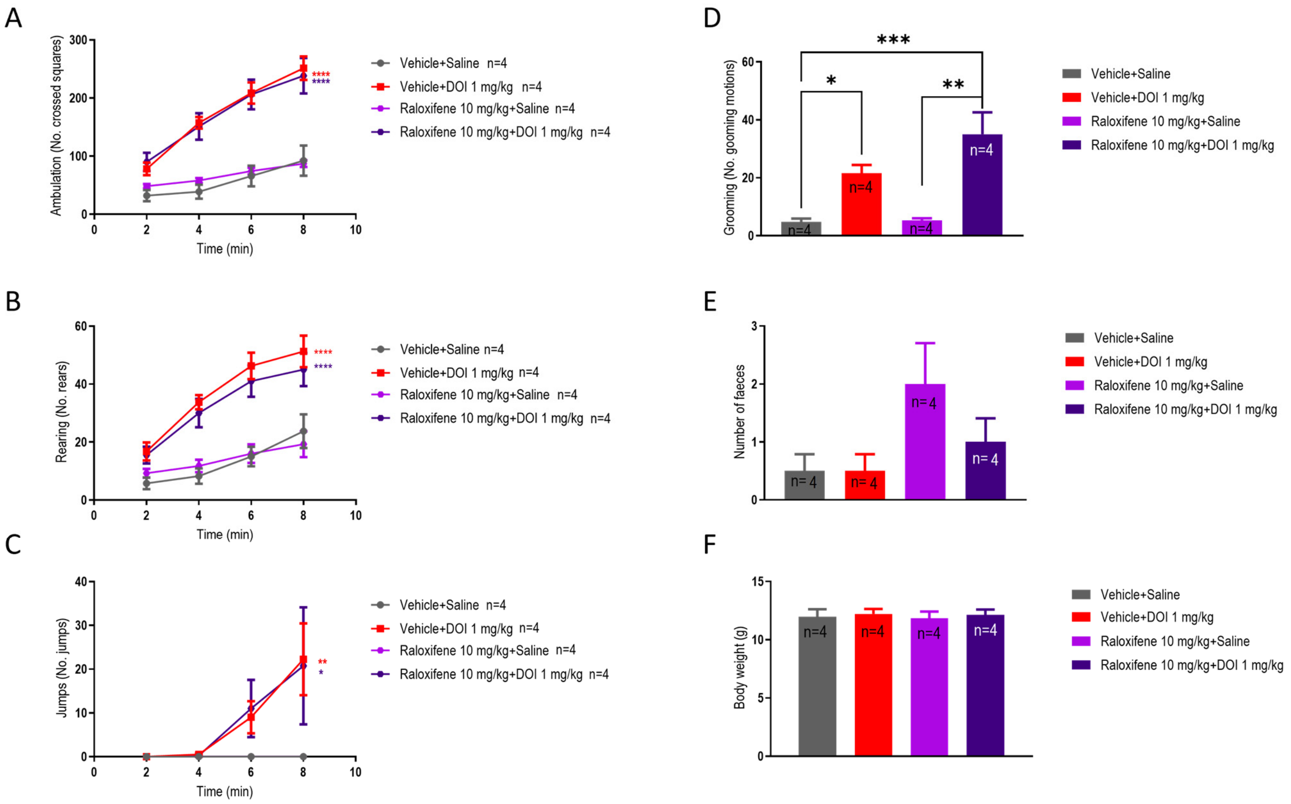

Figure S2. Effects of raloxifene on locomotor activity (ambulation, rearing, jumping), grooming behaviour, faecal output, and body weight in juvenile male mice with or without DOI. The effects of raloxifene on ambulation (**A**), rearing (**B**), jumping (**C**), grooming (**D**), faecal output (**E**), and body weight (**F**). Data are means ± SEM; n = 3-week-old C57BL/6J male mice per group. Experiments were independently repeated to the lowest n. Two-way ANOVA with Bonferroni's correction (* $p = 0.05$; ** $p < 0.01$; *** $p < 0.001$; **** $p < 0.0001$).

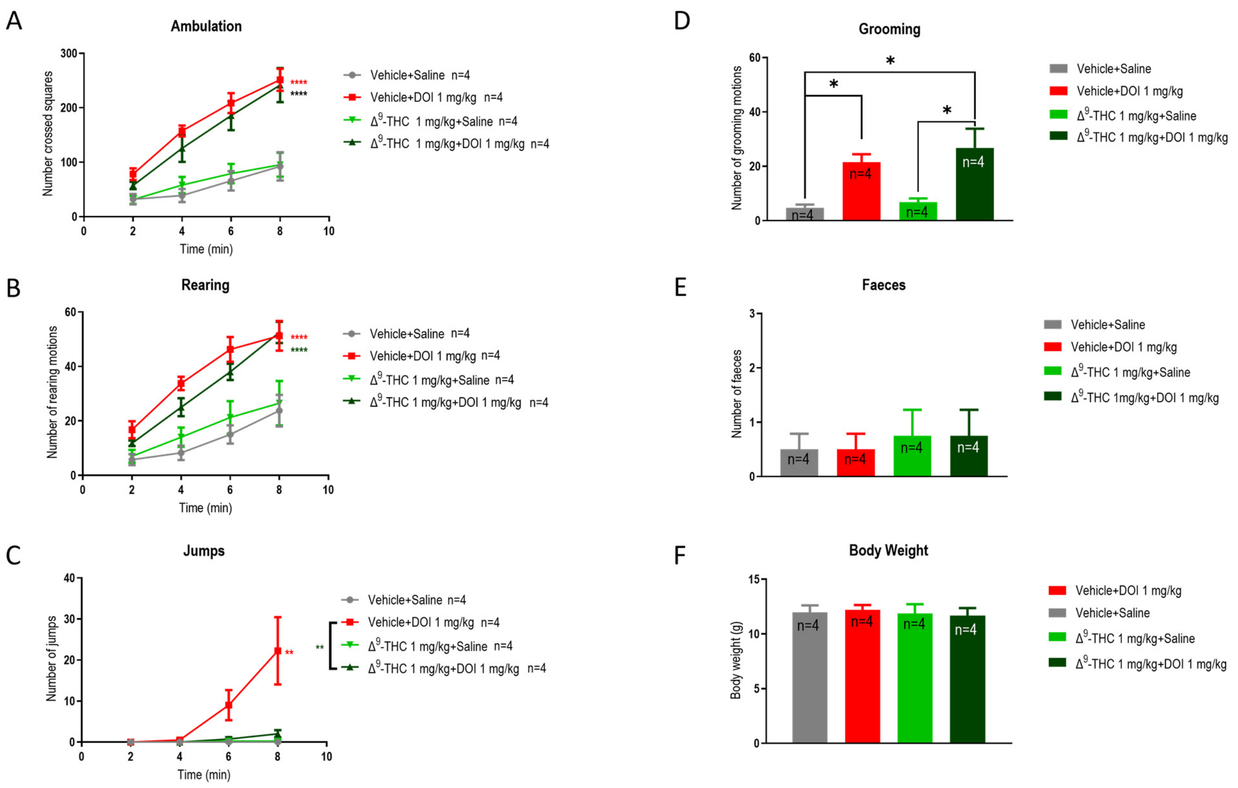

Figure S3. Effects of Δ⁹-THC on locomotor activity (ambulation, rearing, jumping), grooming behaviour, faecal output, and body weight in juvenile male mice with or without DOI. The effects of Δ⁹-THC on ambulation (**A**), rearing (**B**), jumping (**C**), grooming (**D**), faecal output (**E**), and body weight (**F**). Data are means ± SEM; n = 3-week-old C57BL/6J male mice per group. Experiments were independently repeated to the lowest n. Two-way ANOVA with Bonferroni's correction (* $p = 0.05$; ** $p < 0.01$; **** $p < 0.0001$).

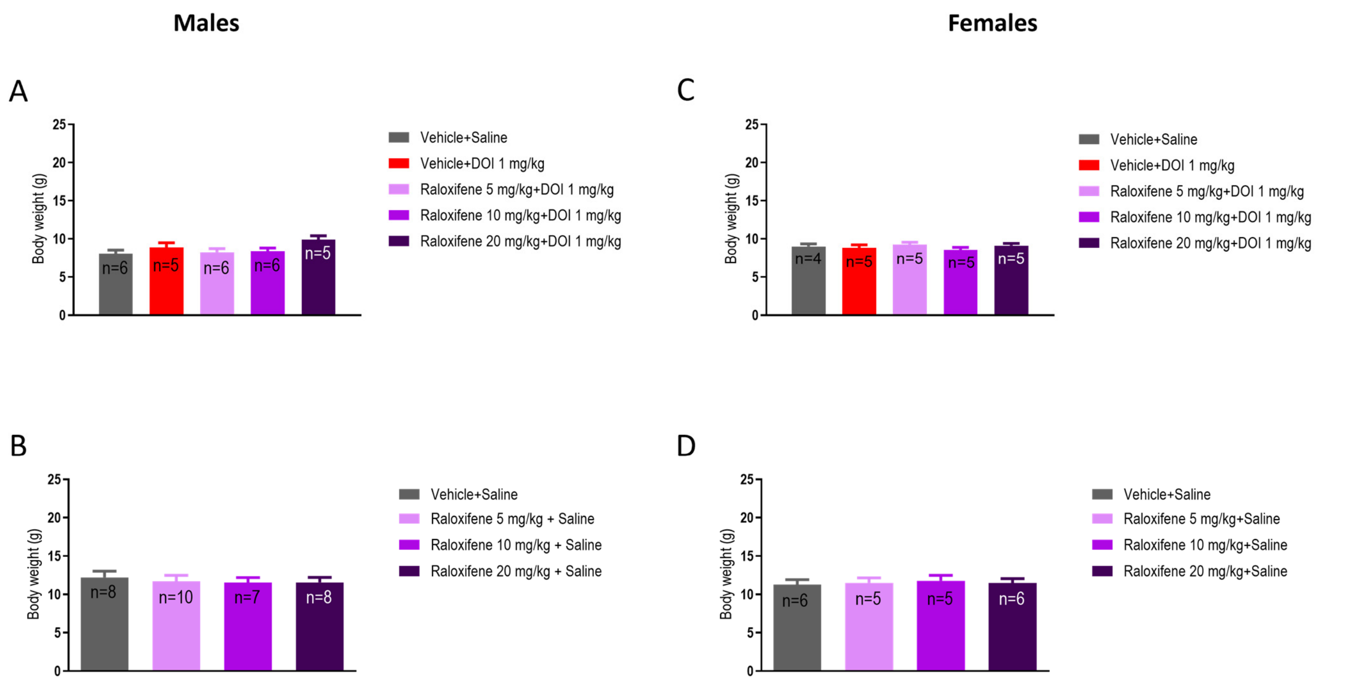

Figure S4. Body weight of juvenile male and female mice with or without DOI. Corresponding to Figures 4 and 5: body weight of male mice injected with raloxifene in the presence (**A**) and absence (**B**) of DOI; female mice in the presence (**C**) and absence (**D**) of DOI. Data are means ± SEM; n = 3-week-old C57BL/6J mice per group. Experiments were independently repeated to the lowest n. Two-way ANOVA with Bonferroni's correction.

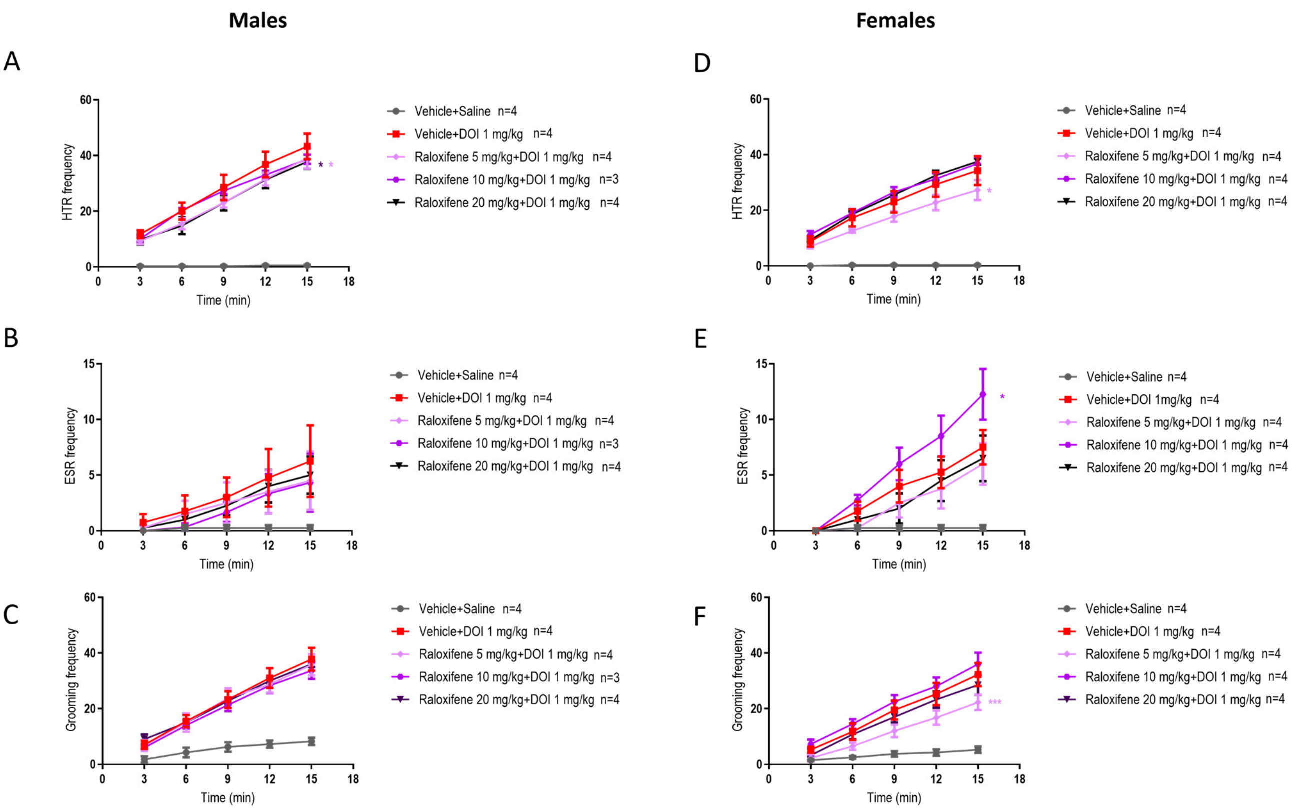

Figure S5. Effects of raloxifene on DOI-induced HTR, ESR, and grooming behaviour in young adult male and female mice. The effects of raloxifene on HTR in males (**A**) and females (**D**); ESR in males (**B**) and females (**E**); grooming in males (**C**) and females (**F**). Data are means ± SEM; n = C57BL/6J mice per group. Experiments were independently repeated to the lowest n. Two-way ANOVA with Bonferroni's post hoc test ($p = 0.05$; * $p < 0.01$; ** $p < 0.001$; *** $p < 0.0001$).

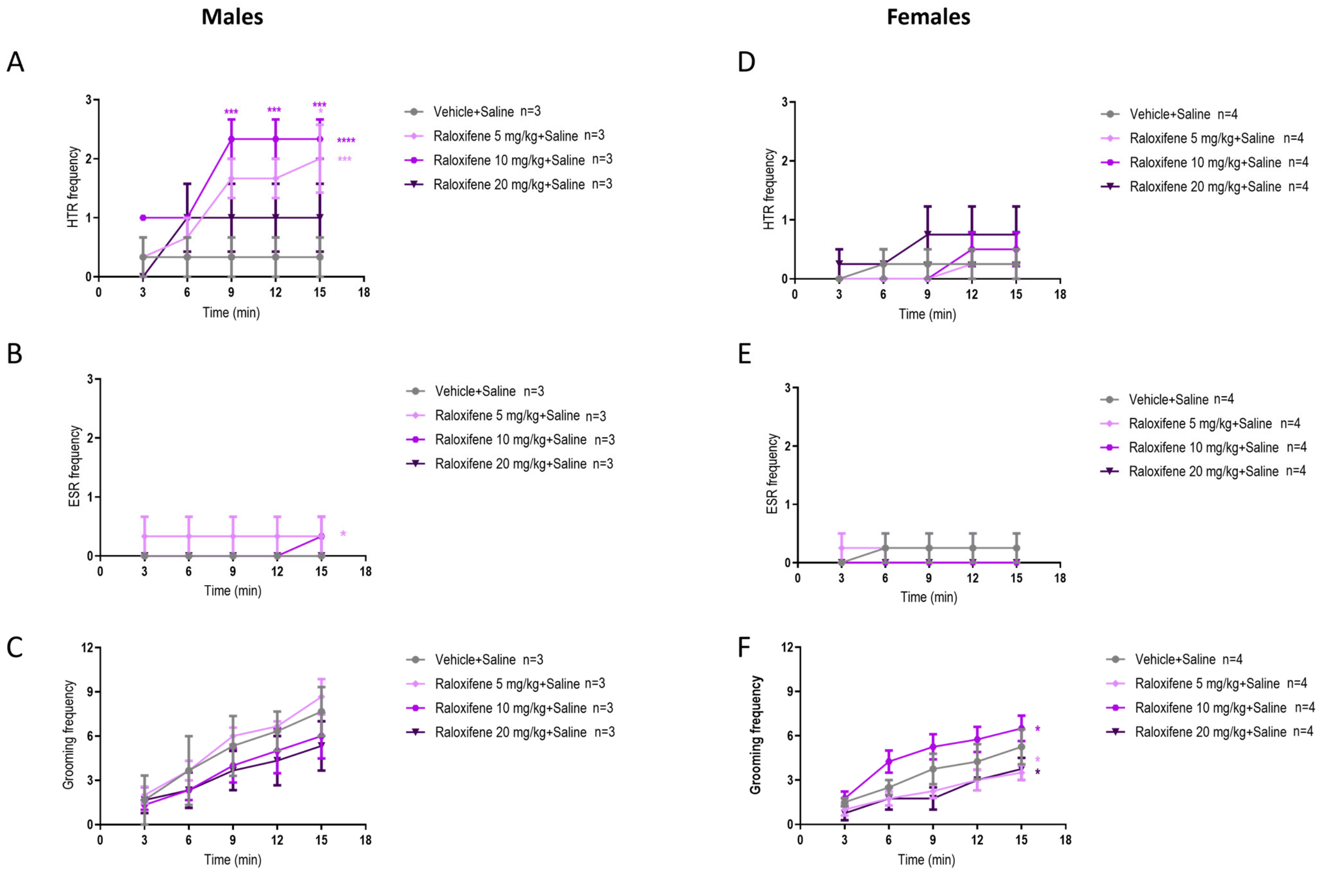

Figure S6. Effects of raloxifene on HTR, ESR, and grooming behaviour in young adult male and female mice in the absence of DOI. The effects of raloxifene on HTR in males (**A**) and females (**D**); ESR in males (**B**) and females (**E**); grooming in males (**C**) and females (**F**). Data are means ± SEM; n = C57BL/6J mice per group. Experiments were independently repeated to the lowest n. Two-way ANOVA with Bonferroni's post hoc test ($p = 0.05$; * $p < 0.01$; ** $p < 0.001$; *** $p < 0.0001$). (* $p = 0.05$).

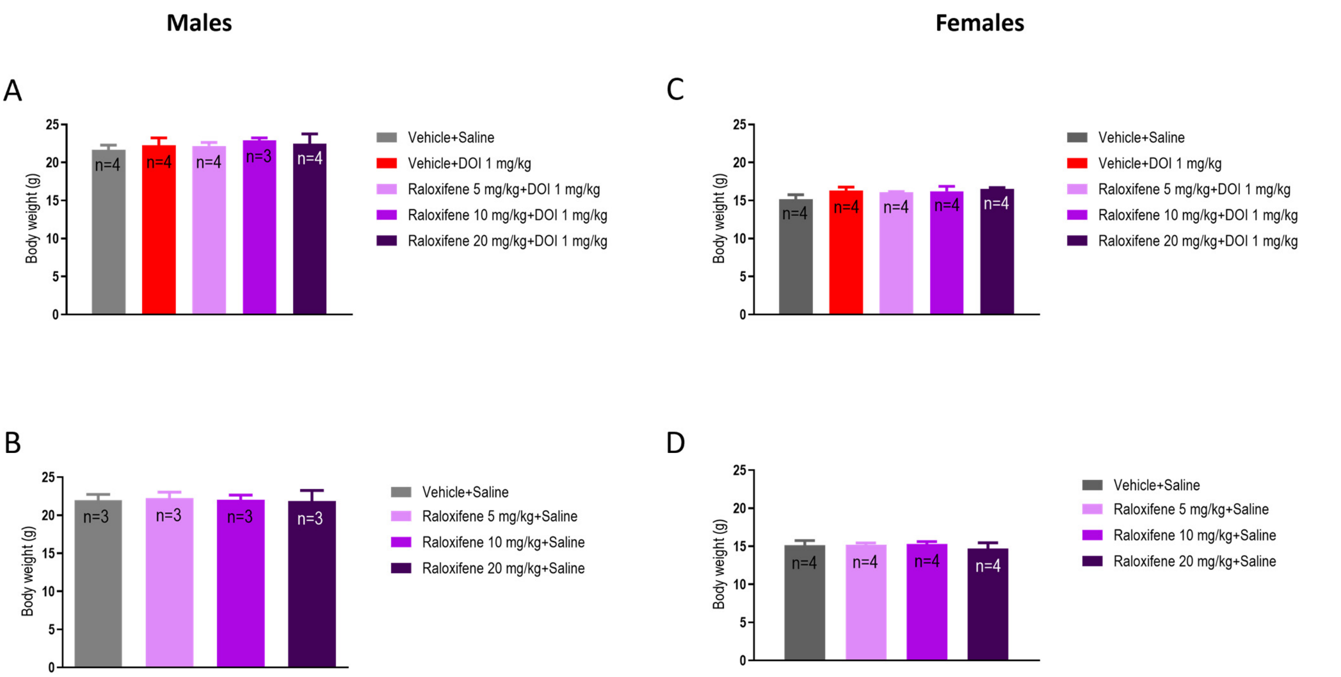

Figure S7. Body weight of young adult male and female mice with or without DOI. Corresponding to Figures A6 and A7: body weight of male mice injected with raloxifene in the presence (**A**) and absence (**B**) of DOI; female mice in the presence (**C**) and absence (**D**) of DOI. Data are means ± SEM; n = C57BL/6J mice per group. Experiments were independently repeated to the lowest n. Two-way ANOVA with Bonferroni's correction
